# Investigating the association of opioid prescription with the incidence of psychiatric disorders: nationwide cohort study in South Korea

**DOI:** 10.1192/bjo.2024.72

**Published:** 2024-05-27

**Authors:** Tak Kyu Oh, Hye Yoon Park, In-Ae Song

**Affiliations:** Department of Anesthesiology and Pain Medicine, College of Medicine, Seoul National University, South Korea; and Department of Anesthesiology and Pain Medicine, Seoul National University Bundang Hospital, South Korea; Department of Psychiatry, Seoul National University Hospital, South Korea; and Department of Psychiatry, College of Medicine, Seoul National University, South Korea

**Keywords:** Big data, depressive disorders, epidemiology, opioid disorders, clinical outcomes measures

## Abstract

**Background:**

The relationship between opioid use and the incidence of psychiatric disorders remains unidentified.

**Aims:**

This study examined the association between the incidence of psychiatric disorders and opioid use.

**Method:**

Data for this population-based cohort study were obtained from the National Health Insurance Service of South Korea. The study included all adult patients who received opioids in 2016. The control group comprised individuals who did not receive opioids in 2016, and were selected using a 1:1 stratified random sampling procedure. Patients with a history of psychiatric disorders diagnosed in 2016 were excluded. The primary end-point was the diagnosis of psychiatric disorders, evaluated from 1 January 2017 to 31 December 2021. Psychiatric disorders included schizophrenia, mood disorders, anxiety and others.

**Results:**

The analysis included 3 505 982 participants. Opioids were prescribed to 1 455 829 (41.5%) of these participants in 2016. Specifically, 1 187 453 (33.9%) individuals received opioids for 1–89 days, whereas 268 376 (7.7%) received opioids for ≥90 days. In the multivariable Cox regression model, those who received opioids had a 13% higher incidence of psychiatric disorder than those who did not (hazard ratio 1.13; 95% CI 1.13–1.14). Furthermore, both those prescribed opioids for 1–89 days and for ≥90 days had 13% (hazard ratio 1.13, 95% CI 1.12–1.14) and 17% (hazard ratio 1.17, 95% CI 1.16–1.18) higher incidences of psychiatric disorders, respectively, compared with those who did not receive opioids.

**Conclusions:**

This study revealed that increased psychiatric disorders were associated with opioid medication use. The association was significant among both short- and long-term opioid use.

Opioids are among the most commonly prescribed analgesics and powerful pain relievers.^1^ However, their use has been linked to an increased risk of dependency and addiction,^2^ leading to a worldwide opioid crisis.^3^ According to a recent study, the risk of opioid misuse and dependency is highest in six nations, namely Australia, Canada, France, Germany, the UK and the USA,^4^ indicating that the opioid crisis is now one of the most serious public health crises.

Psychiatric problems are a worldwide health issue. Depression, drug addiction and schizophrenia affect approximately 120, 90 and 25 million individuals, respectively.^5^ Notably, psychiatric disorders might be closely linked to opioid prescription,^6^ and comorbid psychiatric morbidities are widespread in patients with substance use disorders, including opioid use disorder.^7^ A recent systematic review and meta-analysis of 345 studies reported that people with opioid use disorder have a higher prevalence of depression, anxiety, post-traumatic stress disorder, obsessive–compulsive disorder, panic disorder, bipolar disorder, antisocial personality disorder, borderline personality disorder, attention-deficit/hyperactivity disorder, psychotic disorder and schizophrenia.^8^ However, the study focused on people with opioid use disorder, not all of whom were prescribed opioids. Further, the incidence rate of newly diagnosed psychiatric disorders was not considered in previous studies.^8^ As opioid analgesics are so commonly prescribed, the side-effects of long-term use are important.^1^ Although even short-term use has been linked to the development of psychiatric disorders, no study has yet focused on this issue. Moreover, by using big data with a very large number of cases and controls, it is possible to investigate the association between opioid use and the occurrence of rare psychiatric disorders such as delusional disorder, dissociative and conversion disorders, and tic disorder.

Therefore, we used a nationwide database in South Korea to examine the association between the incidence of psychiatric disorders and opioid use. We also aimed to examine the effect of the prescription period (short or long term) on the association between the incidence of psychiatric disorders and opioid use.

## Method

### Study design and ethical statement

This population-based cohort study adhered to the guidelines of Strengthening the Reporting of Observational Studies in Epidemiology.^9^ The study protocol was exempted from discussion by the Institutional Review Board (IRB) because of the use of publicly available data. The IRB number assigned to this study is X-2307-840-903. The National Health Insurance Service (NHIS) (approval number NHIS-2023-1-115) approved the study protocol, and authorisation was obtained for data access. The IRB waived the need for informed consent because of the use of retrospectively gathered, anonymised data.

### Data source

South Korea's NHIS is the exclusive public health insurance provider. It collects and manages comprehensive data on drug prescriptions, procedures and disease diagnoses. Data were organised and classified according to ICD-10 codes. All Koreans living in South Korea and foreigners who have lived in the country for more than 6 months are required to enrol in the NHIS programme. In the NHIS system, enrolees pay premiums and, in return, receive subsidised treatment or tests from the government, depending on the severity of their illness. Although more than 95% of healthcare providers are private, the government controls 100% of prescriptions, treatments and prices. Therefore, there are no data on the possibility of missing diagnoses or prescriptions registered by doctors. For ICD-10 diagnoses, we considered all diagnoses, not just the primary diagnosis, in this study. Additionally, the NHIS database provides data on socioeconomic indicators and mortality rates of all individuals.^10^

### Study population

**Fig. 1:**
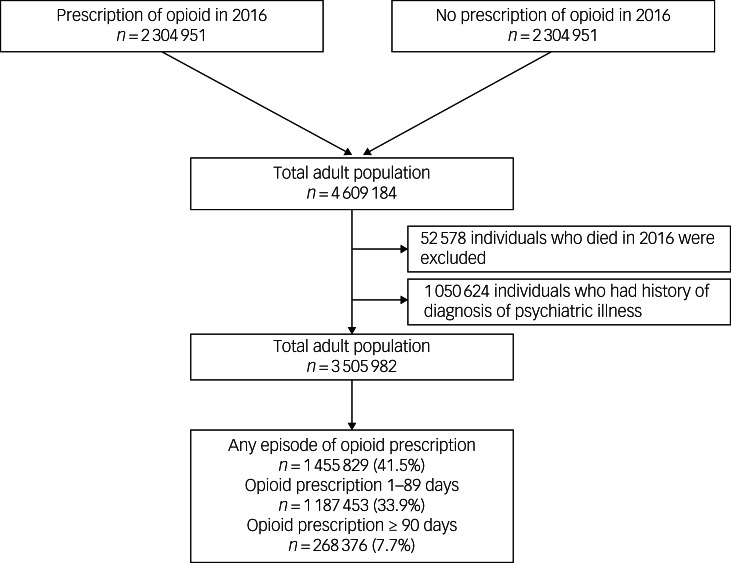
Flow chart depicting the study participant selection process.

We initially requested data extraction for all adult individuals (≥18 years old) who received opioid prescriptions from medical institutions between 1 January 2016 and 31 December 2016. Data were collected for only 1 day of opioid prescription for each individual. Therefore, 2 304 592 adults who received opioid prescriptions in 2016 were included in this study. Afterward, we used a 1:1 stratified random sampling technique, considering age and gender, to request data extraction for 2 304 592 adult individuals in the group who did not receive any opioid prescriptions between 1 January 2016 and 31 December 2016. Therefore, 4 609 184 adults were included in the study. After excluding 52 578 individuals who died in 2016 and 1 050 624 individuals with a history of psychiatric disorder in 2016, 3 505 982 individuals were included in the analysis. Among these, 1 455 829 (41.5%) were prescribed opioids in 2016. Specifically, 1 187 453 (33.9%) and 268 376 (7.7%) individuals were prescribed opioids for 1–89 and ≥90 days, respectively. The participants were classified into three groups: those who did not receive opioids, those prescribed opioids for 1–89 days and those prescribed opioids for ≥90 days, according to the classification criteria for short-term and long-term opioid prescription periods.^11^ The selection process for the study population is illustrated in Fig. 1.

### Study end-point

The primary end-point in this study was the diagnosis of psychiatric disorders, which were evaluated from 1 January 2017 to 31 December 2021. According to a previous study,^12^ psychiatric disorders are classified into four groups: schizophrenia spectrum disorders, mood disorders, anxiety disorders and other psychiatric disorders. The ICD-10 codes for the psychiatric disorders are presented in Supplementary Table 1 available at https://doi.org/10.1192/bjo.2024.72.

### Collected covariates

Demographic information included age and gender assigned at birth. Further, data on the factors associated with the socioeconomic status, household income level and residence of the study population were collected. Household income levels were classified into five groups (medical aid programme groups and four groups according to quartile ratios). Individuals having difficulty in paying insurance premiums because of poverty are classified into a medical aid programme group by the government. The capital and other metropolitan cities were considered urban, and all other areas were classified as rural. Household income and other sociodemographic data are provided directly by NHIS. Because the NHIS charges different premiums based on enrolees’ household income, we collected information about enrolees’ household income, property and residence.

Information regarding underlying disabilities was collected, as all disabilities must be registered in the NHIS database to be eligible to receive various benefits from the social welfare systems in South Korea. All disabilities should be legally determined by a specialist doctor based on the criteria of difficulty in maintaining activities of daily living. Underlying disabilities were categorised according to severity, distinguishing between mild, moderate and severe disabilities. Elixhauser Comorbidity Index calculations for 29 underlying disorders were collected to indicate patients’ comorbid status.^13^ Prescription data for other analgesics, such as paracetamol, nonsteroidal anti-inflammatory drugs, gabapentin and pregabalin, were collected.

### Statistical analysis

Mean values with standard deviations were used to present continuous data, such as age, whereas numbers with percentages were used to display all other categorical variables. Using a *t*-test and chi-squared test, the incidence of psychiatric illnesses in the groups of those who used opioids and those who did not was compared. A multivariable Cox regression model was employed to investigate whether individuals who received opioids exhibited a higher occurrence of psychiatric illnesses than those who did not. This analysis was conducted using the time-to-event methodology. The diagnosis of psychiatric diseases was established as an event. In contrast, the timeframe from 1 January 2017 to the initial diagnosis of psychiatric illnesses was designated as the duration for the time-to-event analysis. The adjusted model incorporated all the variables. An additional multivariable Cox regression model was used to diagnose psychiatric illnesses, with those who received opioids categorised into two distinct subgroups: those who used opioids for 1–89 days and those who used opioids for ≥90 days. This study explored the potential impact of opioid prescription duration on the obtained results. Moreover, multivariable Cox regression models were constructed to examine whether the association among psychiatric illness differed between those who did and did not use opioids, according to the psychiatric illness, in detail. Finally, we performed a sensitivity analysis after excluding 1 231 529 patients who had psychiatric diseases during 2015–2016, because a 1-year timeframe was not sufficient as a washout period. All results are presented as hazard ratios with 95% confidence intervals, and log-log plots were used to verify whether the fundamental assumptions of the Cox proportional hazard models were met. No multicollinearity between variables using the multivariable model was considered at variance inflation factors <2.0. All statistical analyses were conducted with the SPSS program for Windows (version 25.0; IBM Corp., Armonk, New York, USA), and statistical significance was set at *P* < 0.05.

## Results

### Clinicopathological characteristics

The clinicopathological characteristics of the study population (*N* = 3 505 982) are shown in Table 1. Mean age was 53.8 years (s.d. 16.1 years), and 49.9% of the patients (1 750 868 out of 3 505 982) were male. From 1 January 2017 to 31 December 2021, 1 183 310 (33.8%) patients were diagnosed with a psychiatric illness. The incidences of schizophrenia spectrum disorders, mood disorders, anxiety disorders and other psychiatric disorders were 0.9% (32 400 out of 3 505 982), 14.8% (517 194 out of 3 505 982), 20.1% (705 020 out of 3 505 982) and 15.7% (551 882 out of 3 505 982), respectively.

**Table 1:** Clinicopathological characteristics of the study population (*N* = 3 505 982)

Variable	Mean (s.d.) or *n* (%)
Age, years	53.8 (16.1)
Gender, male	1 750 868 (49.9)
Household income level	
Medical aid programme group	97 512 (2.8)
Quartile 1 (lowest)	643 171 (18.3)
Quartile 2	713 606 (20.4)
Quartile 3	863 305 (24.6)
Quartile 4 (highest)	1 127 057 (32.1)
Unknown	61 331 (1.7)
Residence	
Urban area	1 548 906 (44.2)
Rural area	1 957 076 (55.8)
Underlying disability	
Mild to moderate	165 978 (4.7)
Severe	71 505 (2.0)
Underlying comorbidity	
Congestive heart failure	103 372 (2.9)
Cardiac arrhythmias	89 022 (2.5)
Valvular disease	13 925 (0.4)
Pulmonary circulation disorders	6000 (0.2)
Peripheral vascular disorders	296 937 (8.5)
Hypertension, uncomplicated	947 609 (27.0)
Hypertension, complicated	89 871 (2.6)
Paralysis	17 677 (0.5)
Other neurological disorders	71 137 (2.0)
Chronic pulmonary disease	817 153 (23.3)
Diabetes, uncomplicated	467 607 (13.3)
Diabetes, complicated	232 707 (6.6)
Hypothyroidism	130 869 (3.7)
Renal failure	42 348 (1.2)
Liver disease	611 508 (17.4)
Peptic ulcer disease, excluding bleeding	468 895 (13.4)
AIDS/HIV	1792 (0.1)
Lymphoma	4367 (0.1)
Metastatic cancer	21 817 (0.6)
Solid tumour without metastasis	187 251 (5.3)
Rheumatoid arthritis/collagen vascular diseases	140 589 (4.0)
Coagulopathy	26 990 (0.8)
Obesity	3491 (0.1)
Weight loss	16 863 (0.5)
Fluid and electrolyte disorders	121 236 (3.5)
Blood loss anaemia	6064 (0.2)
Deficiency anaemia	138 244 (3.9)
Alcohol use disorder	38 175 (1.1)
Drug use disorder	35 (0.0)
Prescription of other analgesics	
Paracetamol	2 237 197 (25.9)
Non-steroidal anti-inflammatory drugs	908 171 (25.9)
Gabapentin or pregabalin	132 237 (3.8)
Diagnosis of psychiatric illness during 2017–2021	1 183 310 (33.8)
Schizophrenia spectrum disorders	32 400 (0.9)
Mood disorder	517 194 (14.8)
Anxiety disorder	705 020 (20.1)
Other psychiatric illness	551 882 (15.7)

Table 2 displays the findings of the comparison of the clinicopathological features of those who did and those who did not receive opioids. The findings of the comparison of the incidence of psychiatric illnesses among these groups are displayed in Table 3.

**Table 2:** Clinicopathological features of those who did and did not use opioids

Variable	Used opioids, *n* = 1 455 829	Did not use opioids, *n* = 2 050 153
Age, years	54.3 (16.2)	53.1 (16.0)
Gender, male	736 422 (50.6)	1 014 446 (49.5)
Household income level		
Medical aid programme group	46 829 (3.2)	50 683 (2.5)
Quartile 1 (lowest)	269 945 (18.5)	373 226 (18.2)
Quartile 2	305 436 (21.0)	408 170 (19.9)
Quartile 3	367 246 (25.2)	496 059 (24.2)
Quartile 4 (highest)	441 940 (30.4)	685 117 (33.4)
Unknown	24 433 (1.7)	50 683 (2.5)
Residence		
Urban area	609 957 (41.9)	938 949 (45.8)
Rural area	845 872 (58.1)	1 111 204 (54.2)
Underlying disability		
Mild to moderate	79 271 (5.4)	86 707 (4.2)
Severe	26 928 (1.8)	44 577 (2.2)
Underlying comorbidity		
Congestive heart failure	46 161 (3.2)	57 211 (2.8)
Cardiac arrhythmias	39 468 (2.7)	49 554 (2.4)
Valvular disease	5812 (0.4)	8113 (0.4)
Pulmonary circulation disorders	2801 (0.2)	3199 (0.2)
Peripheral vascular disorders	159 983 (11.0)	136 954 (6.7)
Hypertension, uncomplicated	417 680 (28.7)	529 929 (25.8)
Hypertension, complicated	39 658 (2.7)	50 213 (2.4)
Paralysis	5429 (0.4)	12 248 (0.6)
Other neurological disorders	33 229 (2.3)	37 908 (1.8)
Chronic pulmonary disease	421 205 (28.9)	395 948 (19.3)
Diabetes, uncomplicated	214 626 (14.7)	252 981 (12.3)
Diabetes, complicated	105 902 (7.3)	126 805 (6.2)
Hypothyroidism	60 163 (4.1)	70 706 (3.4)
Renal failure	17 106 (1.2)	25 242 (1.2)
Liver disease	299 272 (20.6)	312 236 (15.2)
Peptic ulcer disease, excluding bleeding	256.282 (17.6)	212 613 (10.4)
AIDS/HIV	805 (0.1)	987 (0.0)
Lymphoma	1939 (0.1)	2428 (0.1)
Metastatic cancer	9777 (0.7)	12 040 (0.6)
Solid tumour without metastasis	79 495 (5.5)	107 756 (5.3)
Rheumatoid arthritis/collagen vascular diseases	88 061 (6.0)	52 528 (2.6)
Coagulopathy	12 393 (0.9)	14 597 (0.7)
Obesity	1932 (0.1)	1559 (0.1)
Weight loss	7675 (0.5)	9188 (0.4)
Fluid and electrolyte disorders	60 733 (4.2)	60 503 (3.0)
Blood loss anaemia	2853 (0.2)	3211 (0.2)
Deficiency anaemia	64 456 (4.4)	73 788 (3.6)
Alcohol use disorder	19 219 (1.3)	18 956 (0.9)
Drug use disorder	18 (0.0)	17 (0.0)
Prescription of other analgesics		
Paracetamol	1 445 186 (99.3)	792 011 (38.6)
Non-steroidal anti-inflammatory drugs	467 246 (32.1)	440 925 (21.5)
Gabapentin or pregabalin	98 372 (6.8)	33 865 (1.7)

**Table 3:** Diagnosis of psychiatric illness during 2017–2021 for those who did and did not use opioids

Diagnosis of psychiatric illness during 2017–2021	Used opioids, *n* = 1 455 829	Did not use opioids, *n* = 2 050 153	*P*-value
Total psychiatric illness	575 214 (39.5)	608 096 (29.7)	<0.001
Schizophrenia	13 539 (0.9)	18 861 (0.9)	0.335
Mood disorder	253 380 (17.4)	263 814 (12.9)	<0.001
Anxiety disorder	351 719 (24.2)	353 301 (17.2)	<0.001
Other psychiatric illness	275 917 (19.0)	275 965 (13.5)	<0.001
Diagnosis of psychiatric illness during 2017–2021 in detail			
Schizophrenia	6430 (0.4)	9670 (0.5)	<0.001
Delusional disorders	1917 (0.1)	2755 (0.1)	0.494
Acute and transient psychotic disorders	2040 (0.1)	2589 (0.1)	<0.001
Schizoaffective disorder	802 (0.1)	1040 (0.1)	0.079
Other non-organic psychotic disorders	702 (0.0)	913 (0.0)	0.113
Unspecified non-organic psychosis	3767 (0.3)	5120 (0.2)	0.098
Bipolar disorder	37 132 (2.6)	48 965 (2.4)	<0.001
Major depressive disorder	223 001 (15.3)	227 577 (11.1)	<0.001
Persistent mood disorder	13 525 (0.9)	13 686 (0.7)	<0.001
Unspecified mood disorder	18 649 (1.3)	16 533 (0.8)	<0.001
Phobic anxiety disorders	10 901 (0.7)	10 521 (0.5)	<0.001
Other anxiety disorders	347 398 (23.9)	348 751 (17.0)	<0.001
Substance use disorder	15 194 (1.0)	17 716 (0.9)	<0.001
Obsessive–compulsive disorder	3041 (0.2)	3549 (0.2)	<0.001
Reaction to severe stress, and adjustment disorders	36 132 (2.5)	38 336 (1.9)	<0.001
Dissociative and conversion disorders	515 (0.0)	518 (0.0)	<0.001
Somatoform disorders	83 813 (5.8)	77 420 (3.8)	<0.001
Other non-psychotic mental disorders	81 992 (5.6)	75 799 (3.7)	<0.001
Eating disorders	4075 (0.3)	4617 (0.2)	<0.001
Sleep disorders not resulting from a substance or known physiological condition	118 163 (8.1)	118 121 (5.8)	<0.001
Mental and behavioural disorders associated with the puerperium	27 (0.0)	30 (0.0)	0.371
Other specific personality disorders	875 (0.1)	1134 (0.1)	0.065
Other disorders of adult personality and behaviour	155 (0.0)	130 (0.0)	<0.001
Unspecified disorder of adult personality and behaviour	92 (0.0)	122 (0.0)	0.663
Attention-deficit hyperactivity disorders	1833 (0.1)	2181 (0.1)	<0.001
Tic disorder	1009 (0.1)	1057 (0.1)	<0.001
Mental disorder, not otherwise specified	1051 (0.1)	1362 (0.1)	0.043

The incidence of mood disorders, anxiety disorders and other psychiatric illnesses was significantly higher in the opioid prescription group, with rates of 17.4% (253 380 out of 1 455 829), 24.2% (351 719 out of 1 455 829) and 19.0% (275 917 out of 1 455 829), respectively. In contrast, those who were not prescribed opioids exhibited lower rates of these disorders, at 12.9% (263 814 out of 2 050 153), 17.2% (353 301 out of 2 050 153) and 13.5% (275 965 out of 2 050 153), respectively.

### Opioid use and psychiatric illness

The findings of the multivariable Cox regression model for the diagnosis of psychiatric diseases are shown in Table 4. Those who received opioids had a 14% higher incidence of psychiatric disorders than those who did not (hazard ratio 1.13, 95% CI 1.13–1.14; *P* < 0.001; model 1). Furthermore, those prescribed opioids for 1–89 days and those prescribed opioids for ≥90 days had 14% (hazard ratio 1.14, 95% CI 1.13–1.14; *P* < 0.001; model 2) and 17% (hazard ratio 1.17, 95% CI 1.16–1.18; *P* < 0.001; model 2) higher incidences of psychiatric disorders, respectively, than those who were not prescribed opioids. All other hazard ratios with 95% confidence intervals for the variables are presented in Supplementary Table 2. The results of sensitivity analysis after excluding 1 231 529 patients who had psychiatric diseases during 2015–2016 are displayed in Supplementary Table 3, and were similar to the previous results.

**Table 4:** Multivariable Cox regression model for the diagnosis of psychiatric diseases

Variable	Hazard ratio (95% CI)	*P*-value
Total diagnosis of major psychiatric illness		
Multivariable model 1		
Did not use opioids	1	
Used opioids	1.13 (1.13–1.14)	<0.001
Multivariable model 2		
Did not use opioids	1	
Used opioids (1–89 days)	1.14 (1.13–1.14)	<0.001
Used opioids (≥90 days)	1.17 (1.16–1.18)	<0.001

### Psychiatric illness in detail

The multivariable Cox regression model for psychiatric diseases is presented in Table 5. Those who received opioids showed 16% (hazard ratio 1.16, 95% CI 1.13–1.20; *P <* 0.001), 18% (hazard ratio 1.18, 95% CI 1.17–1.19; *P <* 0.001), 16% (hazard ratio 1.16, 95% CI 1.15–1.16; *P <* 0.001) and 15% (hazard ratio 1.15, 95% CI 1.14–1.15; *P <* 0.001) higher incidences of schizophrenia spectrum disorders, mood disorders, anxiety disorders and other psychiatric illnesses, respectively, than those who did not.

**Table 5:** Multivariable Cox regression model for psychiatric diseases in detail

Variable	Hazard ratio (95% CI)	*P*-value
	Used opioids (versus did not)	
Psychiatric illness in detail (model 1)		
Schizophrenia spectrum disorders	1.16 (1.13–1.20)	<0.001
Mood disorder	1.18 (1.17–1.19)	<0.001
Anxiety disorder	1.16 (1.15–1.16)	<0.001
Other psychiatric illness	1.15 (1.14–1.15)	<0.001
Psychiatric illness in detail (model 2)		
Schizophrenia	1.15 (1.10–1.20)	<0.001
Delusional disorders	1.13 (1.04–1.22)	0.003
Acute and transient psychotic disorders	1.15 (1.07–1.24)	<0.001
Schizoaffective disorder	1.12 (0.99–1.26)	0.075
Other non-organic psychotic disorders	1.19 (1.04–1.36)	0.011
Unspecified non-organic psychosis	1.15 (1.09–1.22)	<0.001
Bipolar disorder	1.15 (1.13–1.17)	<0.001
Major depressive disorder	1.21 (1.20–1.22)	<0.001
Persistent mood disorder	1.13 (1.10–1.17)	<0.001
Unspecified mood disorder	1.25 (1.22–1.29)	<0.001
Phobic anxiety disorders	1.18 (1.14–1.22)	<0.001
Other anxiety disorders	1.20 (1.19–1.21)	<0.001
Substance use disorder	1.20 (1.17–1.25)	<0.001
Obsessive–compulsive disorder	0.99 (0.93–1.05)	0.677
Reaction to severe stress, and adjustment disorders	1.04 (1.02–1.06)	<0.001
Dissociative and conversion disorders	1.20 (1.03–1.40)	0.019
Somatoform disorders	1.18 (1.17–1.19)	<0.001
Other non-psychotic mental disorders	1.21 (1.20–1.23)	<0.001
Eating disorders	1.12 (1.06–1.18)	<0.001
Sleep disorders not resulting from a substance or known physiological condition	1.16 (1.15–1.18)	<0.001
Mental and behavioural disorders associated with the puerperium	0.79 (0.44–1.41)	0.418
Other specific personality disorders	1.05 (0.93–1.18)	0.427
Other disorders of adult personality and behaviour	1.73 (1.25–2.41)	0.001
Unspecified disorder of adult personality and behaviour	1.16 (0.81–1.67)	0.420
Attention-deficit hyperactivity disorders	1.00 (0.93–1.09)	0.940
Tic disorder	1.13 (1.01–1.26)	0.037
Mental disorder, not otherwise specified	1.13 (1.02–1.26)	0.022

## Discussion

Opioid prescriptions were associated with an increased incidence of psychiatric illnesses in this population-based cohort. This relationship was observed with both short- and long-term opioid use. Furthermore, opioid prescriptions were linked to increased incidence of all four psychiatric diseases, including schizophrenia spectrum disorders, mood disorders, anxiety disorders and other mental illnesses. Even for short-term opioid prescriptions, our findings revealed that opioid medication could be linked with newly diagnosed psychiatric disorders.

This study focused on the incidence of psychiatric disorders over 5 years (2017–2021). Prevalence is operationally defined as quantifying disease cases within a designated population during a predetermined timeframe. In contrast, incidence is operationally defined as the measurement of new disease occurrences within a specified population during a particular duration.^14^ Our study differs from previous studies,^8^ as we excluded adult individuals with a history of psychiatric illness and compared new occurrences of psychiatric disorders during 2017–2021 between those who did and did not receive opioids.

Notably, some mechanisms may be related to the relationship between opioid use and the incidence of psychiatric disorders. According to data from preclinical studies, opioids and their receptor systems modulate neural systems that are dysregulated in mood disorders such as major depressive disorder.^15^ Furthermore, opioids influence reward processing and emotional control in rodent models.^15^ Another recent *in vivo* study reported that the opioid system may be involved in neurocircuits linked to individual differences in adult attachment behavior.^16^ Moreover, the findings imply that variations in mu-opioid receptor availability are related to social interactions and psychological well-being, and thus contribute to the development of psychiatric disorders.^16^ However, the neurobiological mechanisms have been derived from preclinical or animal studies.^15^ Future studies are needed to confirm the relationship between opioid usage and psychiatric disorders accurately.

The physical conditions required for the prescription of opioids could also have affected the increased association with psychiatric disorders in this study. Opioids are commonly administered for pain relief in patients with acute, chronic or cancer pain.^17^ Conditions, such as chronic or cancerous pain, are independent associated factors for psychiatric disorders.^18^ Over 50% of patients diagnosed with advanced cancer exhibit symptoms that meet the criteria for psychiatric disorders.^19^ Moreover, cancer pain usually co-occurs with psychiatric disorders such as depression.^20^ Chronic pain often co-occurs with psychiatric illnesses, such as depression and anxiety, and may be associated with a higher frequency and duration of chronic pain.^21^ Recently, researchers have discovered significant overlaps between pain- and depression-induced neuroplastic changes and neurobiological mechanism modifications.^22^ Such an overlap is critical for promoting the occurrence and development of chronic pain and chronic pain-induced depression.^22^

A significant association between short- and long-term opioid prescription and an increased incidence of psychiatric disorders is an important finding of this study. Long-term opioid medication and the increasing duration of opioid prescriptions have been identified as associated factors for depression in previous studies.^23,24^ No study has focused on the association of psychiatric disorders with short-term opioid use. People prescribed opioids for short-term use have a higher chance of developing long-term opioid use,^11^ and the clinical significance of the impact of opioid prescriptions on the incidence of psychiatric disorders might be similar regardless of the opioid prescription period. It is possible that longer-term opioid use is actually related to more chronic physical diseases, which are in turn related to psychiatric disorders. Shorter use may be associated with trauma (e.g. broken leg).^25^ However, the evidence regarding this is insufficient, and further studies are needed.

Psychosocial vulnerability among people who receive opioids should be also considered when interpreting our results. A previous cohort study revealed that many patients with opioid use disorder reported severe psychosocial difficulties, such as unemployment, poor income, food insecurity and the absence of dependable transportation.^26^ Psychosocial vulnerability has been identified as a contributing factor to the development of psychiatric illness, including depression.^27^ Thus, the increased incidence of psychiatric illness in this population may be influenced by their psychosocial vulnerability, according to our findings. This suggests that psychosocial support may also be an essential factor in preventing the development of psychiatric illnesses in people who are prescribed opioids.

This study had some limitations. First, the opioid dose was not considered in this investigation. Second, certain relevant characteristics, such as body mass index, smoking history and alcohol intake, were not included as covariates because of the paucity of information in the NHIS database. Third, as our study used data from a national registration database in South Korea, generalisability to other countries may be restricted. Fourth, residual or unmeasured variables may have influenced our findings. Fifth, some people who were prescribed opioids in 2016 might have discontinued opioid administration during the study period (2017–2021), whereas those who did not use opioids in 2016 might have started opioid administration during this period, which could have affected our results. Sixth, we determined the presence of psychiatric illnesses based on the diagnoses listed in the NHIS database. However, this may be affected by patients’ access to healthcare providers. For example, patients who visit an out-patient clinic for a prescription of opioids for pain management are more likely to be diagnosed with a psychiatric illness at the same time. Finally, while using a very large sample may be advantageous, as it ensures statistical power, caution should be used when determining whether the detected statistical differences actually have clinical significance.

In summary, we conducted a population-based cohort analysis in South Korea and found a significant association between opioid prescriptions and a higher incidence of psychiatric disorders. This relationship was observed in individuals who had used opioids in the short and long term.

## Supporting information

Oh et al. supplementary material 1 Oh et al. supplementary material

Oh et al. supplementary material 2 Oh et al. supplementary material

Oh et al. supplementary material 3 Oh et al. supplementary material

## Data Availability

The data that support the findings of this study are available from the corresponding author, I.-A.S., upon reasonable request.
