# Supplementary material for: Investigating the association of opioid prescription with the incidence of psychiatric disorders: nationwide cohort study in South Korea
Source: BJPsych Open. 2024 May 27;10(3):e122. doi: 10.1192/bjo.2024.72 (PMC11363087; doi:10.1192/bjo.2024.72)
Supplement: Oh et al. supplementary material 2 — Oh et al. supplementary material [file S2056472424000723sup002.docx]

eTable 2. All other HRs with 95% CIs of multivariable model 1

| Variable | | HR (95% CI) | *P*-value |
| --- | --- | --- | --- |
| Age, year | | 1.02 (1.02, 1.02) | <0.001 |
| Sex, male | | 0.78 (0.77, 0.78) | <0.001 |
| Household income level | |  |  |
|  | Medical aid program group | 1.16 (1.15, 1.18) | <0.001 |
|  | Q1 in quartile (lowest) | 1 |  |
|  | Q2 in quartile | 0.99 (0.98, 0.99) | <0.001 |
|  | Q3 in quartile | 0.97 (0.97, 0.98) | <0.001 |
|  | Q4 in quartile (highest) | 0.96 (0.96, 0.97) | <0.001 |
|  | Unknown | 0.98 (0.97, 1.10) | 0.054 |
| Residence | |  |  |
|  | Urban area | 1 |  |
|  | Rural area | 1.07 (1.07, 1.08) | <0.001 |
| Underlying disability | |  |  |
|  | Mild to moderate | 1.12 (1.11, 1.13) | <0.001 |
|  | Severe | 1.20 (1.18, 1.21) | <0.001 |
| Underlying comorbidity | |  |  |
|  | Congestive heart failure | 1.09 (1.08, 1.10) | <0.001 |
|  | Cardiac arrhythmias | 1.11 (1.10, 1.12) | <0.001 |
|  | Valvular disease | 1.10 (1.09, 1.11) | <0.001 |
|  | Pulmonary circulation disorders | 1.09 (1.05, 1.13) | <0.001 |
|  | Peripheral vascular disorders | 1.11 (1.10, 1.11) | <0.001 |
|  | Hypertension, uncomplicated | 1.03 (1.02, 1.03) | <0.001 |
|  | Hypertension, complicated | 0.99 (0.98, 1.00) | 0.059 |
|  | Paralysis | 1.20 (1.17, 1.23) | <0.001 |
|  | Other neurological disorders | 1.25 (1.23, 1.26) | <0.001 |
|  | Chronic pulmonary disease | 1.15 (1.14, 1.15) | <0.001 |
|  | Diabetes, uncomplicated | 1.03 (1.02, 1.03) | <0.001 |
|  | Diabetes, complicated | 1.03 (1.02, 1.04) | <0.001 |
|  | Hypothyroidism | 1.04 (1.03, 1.05) | <0.001 |
|  | Renal failure | 1.11 (.10, 1.13) | <0.001 |
|  | Liver disease | 1.09 (1.08, 1.09) | <0.001 |
|  | Peptic ulcer disease, excluding bleeding | 1.15 (1.14, 1.15) | <0.001 |
|  | AIDS/HIV | 1.05 (0.97, 1.13) | 0.241 |
|  | Lymphoma | 1.12 (1.07, 1.17) | <0.001 |
|  | Metastatic cancer | 1.32 (1.30, 1.35) | <0.001 |
|  | Solid tumor without metastasis | 1.10 (1.09, 1.11) | <0.001 |
|  | Rheumatoid arthritis/collagen vascular diseases | 1.06 (1.06, 1.07) | <0.001 |
|  | Coagulopathy | 1.07 (1.05, 1.09) | <0.001 |
|  | Obesity | 1.14 (1.09, 1.20) | <0.001 |
|  | Weight loss | 1.19 (1.16, 1.22) | <0.001 |
|  | Fluid and electrolyte disorders | 1.13 (1.12, 1.14) | <0.001 |
|  | Blood loss anemia | 1.07 (1.03, 1.11) | 0.001 |
|  | Deficiency anemia | 1.06 (1.05, 1.07) | <0.001 |
|  | Alcohol use disorder | 1.17 (1.15, 1.19) | <0.001 |
|  | Drug use disorder | 1.50 (1.29, 1.73) | <0.001 |
| Prescription of other analgesics | |  |  |
|  | Paracetamol | 1.20 (1.19, 1.20) | <0.001 |
|  | NSAIDs | 1.12 (1.12, 1.12) | <0.001 |
|  | Gabapentin or pregabalin | 1.20 (1.19, 1.20) | <0.001 |

HR, hazard ratio; CI, confidence interval; AIDS, Acquired immunodeficiency syndrome; HIV, human immunodeficiency virus; NSAIDs, Nonsteroidal anti-inflammatory drugs
