# Supplementary material for: Investigating the association of opioid prescription with the incidence of psychiatric disorders: nationwide cohort study in South Korea
Source: BJPsych Open. 2024 May 27;10(3):e122. doi: 10.1192/bjo.2024.72 (PMC11363087; doi:10.1192/bjo.2024.72)
Supplement: Oh et al. supplementary material 3 — Oh et al. supplementary material [file S2056472424000723sup003.docx]

eTable 3. Multivariable Cox regression model for the diagnosis of psychiatric diseases after excluding 1,231,529 patients who had psychiatric diseases during 2015-2016

| Variable | | HR (95% CI) | *P*-value |
| --- | --- | --- | --- |
| Total diagnosis of major psychiatric illness | |  |  |
| Multivariable model 1 | |  |  |
|  | Non-users | 1 |  |
|  | Opioid user | 1.11 (1.10, 1.12) | <0.001 |
| Multivariable model 2 | |  |  |
|  | Non-users | 1 |  |
|  | Opioid user (1-89 days) | 1.12 (1.11, 1.13) | <0.001 |
|  | Opioid user (≥ 90 days) | 1.16 (1.16, 1.17) | <0.001 |

HR, hazard ratio; CI, confidence interval
